# Canine lung carcinoma—A descriptive review

**DOI:** 10.3389/fvets.2024.1464659

**Published:** 2025-01-20

**Authors:** Aleksandra Marcinowska, Rodrigo Dos Santos Horta, Felisbina Queiroga, Antonio Giuliano

**Affiliations:** ^1^University Centre of Veterinary Medicine, University of Agriculture Kraków, Kraków, Poland; ^2^Przychodnia Weterynaryjna OnkolVet, Opole, Poland; ^3^Department of Veterinary Medicine and Surgery, Veterinary School, Universidade Federal de Minas Gerais (UFMG), Belo Horizonte, Minas Gerais, Brazil; ^4^Animal and Veterinary Research Centre (CECAV), University of Tras-os-Montes and Alto Douro, Vila Real, Portugal; ^5^Associate Laboratory for Animal and Veterinary Sciences (AL4AnimalS), University of Tras-os-Montes and Alto Douro, Vila Real, Portugal; ^6^Veterinary Medical Centre, City University of Hong Kong, Kowloon, Hong Kong SAR, China

**Keywords:** lung, cancer, carcinoma, pulmonary, canine

## Abstract

Primary lung cancer is a relatively uncommon tumor in dogs, accounting for about 1% of all canine malignancies. Clinical signs in affected dogs can range from being asymptomatic to exhibiting chronic cough, and in rare cases, dyspnoea. Surgical excision of the primary tumor, along with resection of the affected locoregional lymph nodes is the preferred treatment option for most cases. Although chemotherapy, targeted therapy and radiation therapy have been employed, their effectiveness remain controversial. Dogs with stage T1 tumors can experience extended survival times of up to 2 years. However, the prognosis for dogs with advanced metastatic tumors is generally very poor. This review article discusses the etiology, clinical signs, diagnosis, staging, treatment options, and prognosis of primary lung carcinoma in dogs.

## Introduction

### Etiology and clinical signs

Compared to human, primary lung cancer is relatively rare in dogs (1, 2), representing ~1% of all canine tumors (3, 4). These tumors are predominantly malignant however benign lesions, such as adenomas and papillomas, hemangiomas, and granulomas have been reported as well (5, 6) representing a small percentage of dogs seen, with adenoma constituting around 3% of the cases. Approximately 85% of malignant primary pulmonary tumors in dogs are epithelial in origin. The most common types among these are pulmonary papillary and bronchoalveolar carcinomas, both forms of adenocarcinoma, comprising around 32 and 13%, respectively, which can be differentiated or non-differentiated (7–9). Within the adenocarcinoma category, other less frequent types include unclassified (around 17%), tubulopapillary (nearly 2%), mixed (nearly 2%), minimally invasive (0.6%), lepidic (0.6%), solid (0.3%), and acinar carcinomas (0.3%). Additionally, other epithelial lung tumors in dogs include adenosquamous (over 4%), squamous (1.8%), bronchial (0.9%), anaplastic (0.9%), and clear cell carcinomas (0.3%). The literature also notes various other tumor types, such as localized histiocytic sarcomas (5.9%), fibrosarcomas (0.3%), chondrosarcomas (0.3%), osteosarcomas, haemangiopericytomas, mast cell, and neuroendocrine tumors (1.5%) (10–13). Out of these “other” tumor types, histiocytic sarcomas are most frequently diagnosed, especially in Bernese Mountain dogs and Miniature Schnauzers, localizing in the right hemithorax (with pretty much equal distribution between the right middle and right cranial lung lobe). However, this review will focus on the canine pulmonary carcinomas.

The average age of dogs diagnosed with primary lung tumors is around 11 years. However, dogs with anaplastic carcinoma are typically younger, with an average age of 7.5 years (3, 14, 15). Certain dog breeds, including Boxers, Dobermans, Australian Shepherds, Irish Setters, and Bernese Mountain Dogs, brachycephalic and small breed dogs could have an increased risk of developing these types of tumors (3, 14, 16).

In contrast to humans, where a strong association between tobacco exposure and lung carcinoma development has been identified and described, no such factor has been recognized in dogs. Although researchers have investigated potential associations between canine lung carcinoma and environmental factors such as passive smoke exposure and urban living, these correlation have not been conclusively proven (17). However, there is evidence suggesting an increased risk of lung cancer in dogs related to higher levels of anthracosis, which is found in polluted air. This link has been supported by studies assessing bronchoalveolar lavage fluid from dogs with primary lung cancer, where the presence of anthracosis was confirmed (18, 19). The mutations, overexpression, or phosphorylation of the epidermal growth factor receptor (EGFR) as well as other factors might have a role in the etiology of canine pulmonary adenocarcinoma and this will be further described in the Diagnosis section.

Pulmonary carcinoma in dogs typically presents as a large solitary mass. The tumor progression often involves metastasis to the tracheobronchial lymph node, followed by intraparenchymal lung metastases, and more rarely, distant organ metastases. The most common sites for distant metastases are the bone, brain and skin (16). Approximately one-third of dogs with pulmonary neoplasia are diagnosed incidentally during routine geriatric evaluations (3, 8, 20). For those exhibiting clinical signs, cough is the most prevalent, noted in 52–93% of the cases (3, 7, 8, 21, 22). Other symptoms can include dyspnoea in 6–24% of dogs, lethargy in 12–18%, hyporexia in 13%, weight loss in 7–12%, hemoptysis in 3-9%, and lameness in 4% of cases. The latter is often associated with hypertrophic osteopathy (HO) or metastatic lytic bone lesions (3, 7, 8, 21, 23).

Clinical examination findings in dogs with pulmonary carcinoma can often be subtle, primarily affecting the respiratory tract. On thoracic auscultation, an increased bronchovesicular sounds may be present in cases with extensive pulmonary involvement (21). Additionally, a build-up of pleural effusion in these patients can result in dull lung and heart sounds (8). While relatively rare, lameness is often a manifestation of HO, a paraneoplastic syndrome linked to both primary and metastatic lung tumors, though it has also been reported in non-malignant conditions. Dogs with HO due to lung tumors typically exhibit pronounced lameness accompanied by significant clinical abnormalities, such as limb swelling and ocular signs. The pathophysiology behind HO is not fully understood but it involves increased peripheral blood flow, proliferation of vascular connective tissue and bone spicule formation (24–29).

### Staging

Clinical staging of canine primary lung tumors is crucial for treatment decision-making as it conveys valuable prognostic and treatment planning information (30). Up to recently, staging has been based on Owen's TNM classification, first established and published in 1980 (Table 1) (31). However, more recently, Lee et al. (32) have suggested adopting a human-derived lung cancer staging system, which incorporates both clinical staging and the TNM system (Table 2). This modified classification reflects the advancement of imaging techniques with more sensitive diagnostic tools. The new classification has demonstrated prognostic value, more accurately reflecting the severity of tumor burden (32, 33). Unlike Owen's system, which lacks stage grouping and shows inconsistent associations with survival, the human-derived system indicates a clear pattern of decreasing survival times from stages I to IV: 952, 658, 158, and 52 days, respectively (32, 34). It is surprising to see the dogs with stage II pulmonary carcinomas survive over 600 days according to this staging system, however stage II tumors seem to be quite a heterogenous group, including T2 and T3 tumors without lymph node metastasis and also T1-T2 tumors with lymph node metastasis. Although promising, the prognostic significance of this new classification system was initially based on a single study involving 71 dogs, necessitating further validation. This validation was subsequently conducted by McPhetridge et al. (35) who assessed 340 dogs with lung cancer and confirmed the efficacy of this stage classification in predicting outcomes.

**Table 1:** Owen's TNM classification of lung tumors in dogs.

**Clinical stages (TNM) of tumors of the larynx, trachea and lungs—WHO** **(****31****)**	
Primary tumor (T)	T1: ≤ 1 cm, solitary
	T2: Multiple tumors of any size
	T3: Tumor invading neighboring tissues
Regional lymph nodes (N)	N0: No evidence of regional lymph node involvement
	N1: Bronchial lymph node involved.
	N2: Distant lymph node involved
Distant metastasis	M0: No evidence of distant metastasis
	M1: Distant metastasis detected
No stage grouping is proposed.	

**Table 2:** Human-derived lung cancer staging system by Lee et al.

**Human-derived lung cancer staging system** **(****32****)**				
Primary tumor (T)	T1	≤ 3 cm	Solitary	None
	T2	3–5 cm	Solitary	Visceral pleura and main bronchi (not carina)
	T3	5–7 cm	Separate nodule(s) in the same lobe	Chest wall, pericardium, phrenic nerve
	T4	>7 cm	Separate nodule (s) in ipsilateral lung lobe	Mediastinum
Regional lymph nodes (N)	N0: No lymph node metastasis			
	N1: Ipsilateral tracheobronchial lymph node metastasis			
	N2: Distant lymph node metastasis			
Distant metastasis (M)	M0: No distant metastasis			
	M1: Malignant effusion, contralateral lung lobe metastasis, extra-thoracic metastasis			
Stage 1	T1, N0, M0			
Stage 2	T2-3, N0, M0; T1-2, N1, M0			
Stage 3	T4, N0, M0; T3-4, N1, M0; T1-4, N2, M0			
Stage 4	T1-4, N1-2, M1			

## Diagnosis

**Figure 1:**
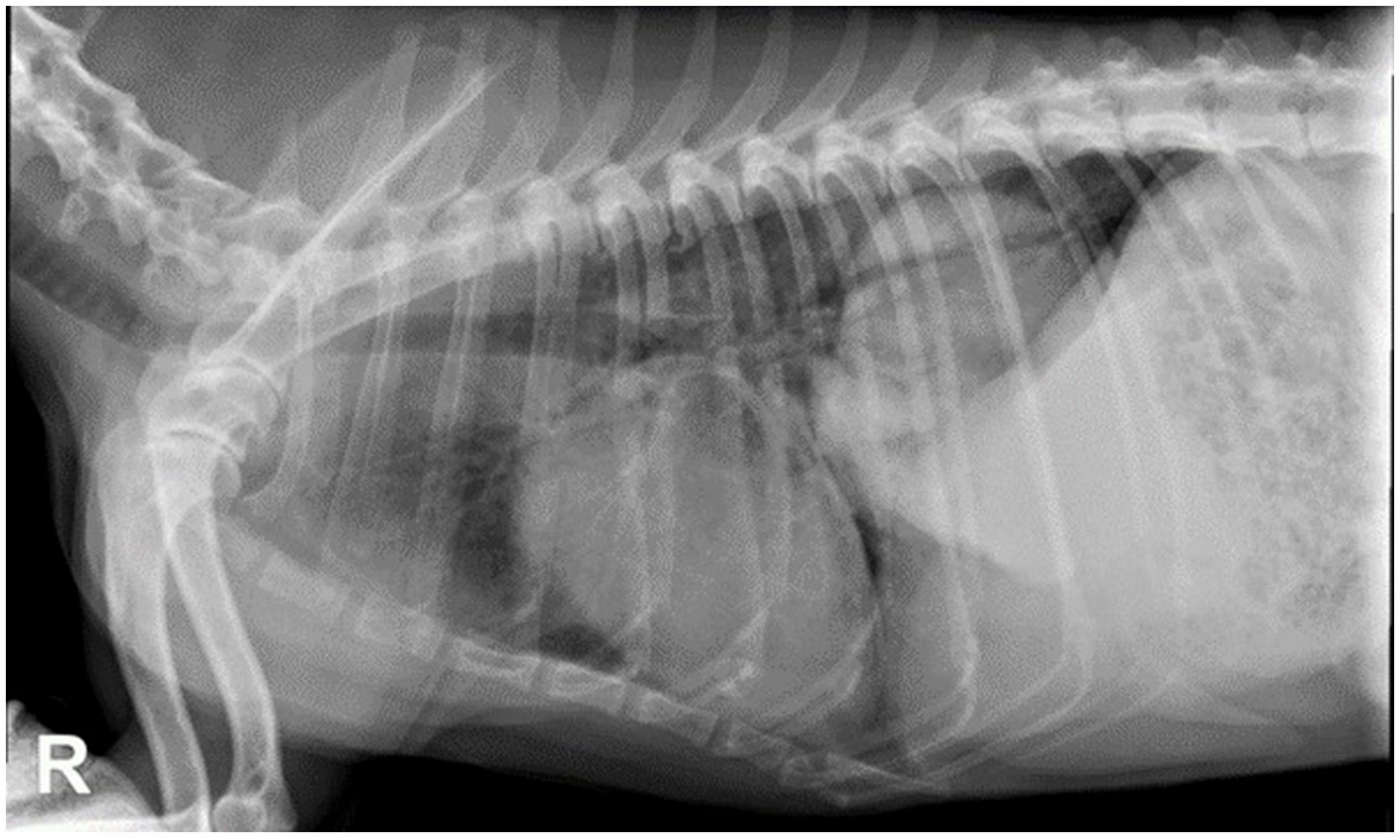
Thoracic radiographies, right lateral view of a 10-year-old mixed breed dog with lung carcinoma. There is a large and well-defined soft tissue opacity in the left caudal lung lobe.

Suspected pulmonary carcinoma is often diagnosed using thoracic radiography. Approximately 80% of pulmonary tumors are usually diagnosed on thoracic radiographs (Figure 1) (7, 8). Radiography is a simple, low-cost, and widely available imaging technique that usually does not require general anesthesia for the patient.

Single tumors are found in the majority of dogs (54–87%), while multiple lesions account for the remainder (13–37%) (7, 21). About 24–50% of tumors have been reported in the left lung, and 50–70% in the right lung (3, 7). Distinguishing pulmonary tumors in the cranial lung lobe from masses affecting the cranial mediastinum can sometimes be challenging on thoracic radiographs. This difficulty arises due to the complex spatial relationship between the mediastinum and surrounding pulmonary parenchyma, and the spatial contrast resolution limitations of two-dimensional radiography (36). Pulmonary masses are generally found lateral to midline with distinct margins contrasted by adjacent gas-filled lungs. Still, medially located pulmonary masses or mediastinal masses that deviate away from the midline can be easily misclassified (36). In a study by Ruby et al. (37) involving 75 dogs and cats, the radiographic differentiation of mediastinal vs. pulmonary masses was assessed. The study also aimed to identify correlations with specific radiographic findings. The overall agreement between radiographs and CT was moderate for both mediastinal (68.6%) and pulmonary masses (63%). Inter-observer agreement was moderate, with moderate to strong intra-observer agreement. Mediastinal masses were significantly more likely to displace other mediastinal structures. Masses located lateral to midline and in the caudal thorax were significantly correlated with a pulmonary origin (37). This knowledge is crucial for generating an appropriate list of differential diagnoses and planning the therapeutic approach. Attempts have been made to use radiographic features to distinguish between different tumor types. Barrett et al. (38) compared pulmonary adenocarcinoma, bronchoalveolar carcinoma, and histiocytic sarcoma (HS). In this study, HS were significantly larger than carcinomas and were more commonly found in the right middle (43%) and left cranial (38%) lung lobes. Additionally, 57% of dogs with HS had internal air bronchograms. Adenocarcinomas were more often found in the left caudal lung lobe (29%) (38).

**Figure 2:**
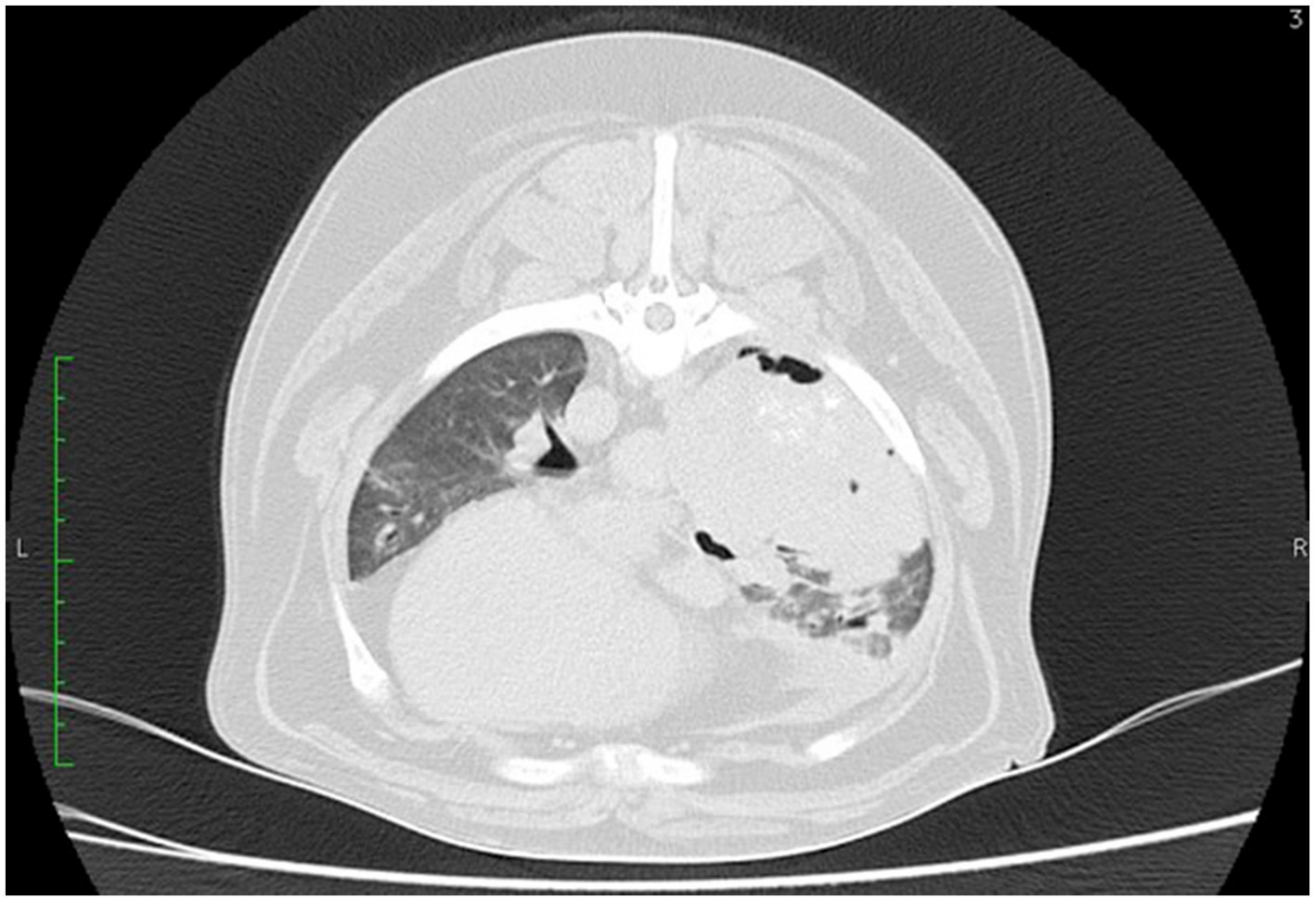
CT scan of the thorax of a 12-year-old mixed breed dog. A large soft tissue attenuating mass is visible on the right lung lobe.

Nowadays, thoracic computed tomography (CT) is a popular diagnostic imaging tool for the general and preoperative assessment of patients with pulmonary neoplasia (Figure 2). CT has become more widely available and plays an increasing role in the diagnosis and staging of thoracic diseases in small animals (39). There are several characteristics of solitary lung tumors that are better appreciated on thoracic CT compared to radiographs in most canine patients. The majority of the tumors are solitary, well-circumscribed, located in a cranial or caudal lobe, and positioned from the center to the periphery of the lobe with internal air bronchograms (40). Study by Nemanic et al. (41) demonstrates CT's superior use to conventional radiographs. In this study 18 dogs with various tumors metastatic to the lungs (including two cases of bronchoalveolar carcinoma) had their lungs simultaneously assessed by thoracic radiographs and thoracic CT. Only 9% of nodules detected on the CT imaging were also visible on the thoracic radiographs (41). CT was able to detect pulmonary nodules as small as 1 mm in diameter, whereas nodules smaller than 7–9 mm were not seen on the radiographs.

These findings are in contrast with the findings from the study by Armbrust et al. (42), in which 33 dogs with various malignant neoplasia had their lungs assessed by thoracic radiographs and CT. In this study, 41% of the nodules seen on the CT were also visible on thoracic radiographs. Interestingly, the smallest nodule size detectable on thoracic radiographs was 3 mm in diameter, whereas for the CT it was 2 mm. The sensitivity of radiography ranged from 71 to 95%, and specificity ranged from 67 to 92%. Radiography had a positive predictive value (PPV) of 83–94%, and a negative predictive value (NPV) of 65–89%, making it an optimal imaging technique for staging Armbrust et al. (42), concluded that thoracic evaluation by CT should be recommended for all large-breed and giant-breed dogs, especially when the detection of pulmonary nodules will have influenced treatment decisions. In another study by Alexander et al. (42), CT was able to detect a greater number of pulmonary nodules compared to computed and film-screen radiography. In addition, CT was better at identifying smaller nodules and was associated with greater diagnostic confidence, observer accuracy, and agreement (43).

A recent study by Able et al. (44) used CT radioman data and features, specifically tumor shape and first-order CT features, from canine primary lung tumors to assess the association with histopathologic characteristics or outcomes following tumor removal. Sixty-seven tumors from 65 dogs were evaluated, with 56 classified as primary pulmonary adenocarcinomas and 11 as non-adenocarcinomas. CT-derived tour variables prognostic for outcomes included volume, maximum axial diameter, and four radioman features: integral total, integral total mean ratio, total Hounsfield unites (HU) and max mean HU ratio. Two distinct features, mean HU ratio and median mean HU ratio, were significantly higher in pulmonary adenocarcinomas compared to non-adenocarcinomas. For dogs with adenocarcinoma, the completeness of excision was associated with overall survival, while a higher mitotic index and histologic score were associated with shorter disease-free survival. Tumor volume was also significantly associated with tumor invasion (44). While texture and shape analysis on CT could provide diagnostic and prognostic information, further studies are needed.

Thoracic CT is also considered more sensitive and accurate than thoracic radiographs in the assessment of tracheobronchial lymph node (TBLN) metastasis. In a study by Paolini et al. (45) evaluating 14 dogs with pulmonary tumors, the accuracy of CT for detecting metastasis to TBLN was 93%, compared with 57% for thoracic radiography. The sensitivity of CT for detecting lymph node metastasis was 83%, compared with 0% for radiographs. Specificity for both CT and radiography was 100%, as no false-positive results were identified with either imaging technique. The PPV for CT was 100%, compared with 0% for radiography, and the NPV for CT was 89%, compared with 57% for radiography (45). In another study that assessed CT characteristics of TBLN metastasis, 16 out of 18 dogs diagnosed with primary pulmonary tumor were compared with 10 normal dogs. The study concluded that metastatic disease in the TBLN was significantly more likely when a transverse maximum lymph node diameter of 12 mm or lymph node-to-thoracic body ratio of 1.05 were used as cutoffs. In addition, lymph node heterogeneity and/or ring contrast enhancement patterns were significantly related to metastatic disease (46).

Thoracic ultrasound may also be used to assess pulmonary neoplasia and is a valuable tool for obtaining tissue samples via fine needle aspirate (FNA) or tru-cut biopsy. Ultrasonographically, pulmonary masses may appear hypoechoic or exhibit variable echogenicity and tumors generally lack discernible bronchi and normal branching vessels (47). Due to some animals presenting with respiratory distress, lung ultrasound, which can be performed quickly in a conscious dog in a standing or sternal position, has gained interest in recent years and seems to be the method of choice for these patients.

Łobaczewski et al. (48) assessed 66 dyspneic dogs from whom cardiac pathology was excluded. They developed and evaluated a diagnostic algorithm for lung ultrasound using a Classification and Regression Tree (CART) to differentiate between lung neoplasm (LN) and bacterial pneumonia (BP) in dyspneic dogs without heart disease. The algorithm was based on three lung ultrasound (LUS) abnormalities: tumor, hepatization, and subpleural consolidations. This method allows for distinguishing between bacterial pneumonia and lung neoplasm in dyspneic dogs with a high probability of a conclusive result (91%) and high accuracy (>95%) (48).

Pulmonary masses on ultrasound may appear hypoechoic or exhibit mixed echogenicity. Tumors are considered to have both lack discernible bronchi and normal branching vessels (49, 50). Pacholec et al. (51) evaluated 62 dogs for respiratory signs or pulmonary metastatic neoplasia screening using thoracic ultrasound, thoracic radiographs, and thoracic CT. For thoracic radiographs, the sensitivity and specificity were 64 and 73%, respectively, with positive likelihood ratio (LR+) and negative likelihood ratio (LR-) of 2.37 and 0.49. For thoracic ultrasound, the sensitivity and specificity were 60 and 65%, with LR+ and LR- of 1.71 and 0.62, respectively. The study results indicate that thoracic ultrasound had similar sensitivity to thoracic radiography, with both modalities missing nodules when used for screening. The low specificity and NPV suggest that caution should be used when assuming thoracic radiographs and thoracic ultrasound to rule out the presence of secondary nodules (51).

The use of contrast-enhanced ultrasonography (CEUS) has also been described and utilized in veterinary patients with pulmonary lesions. As mentioned earlier, differentiating between lung and mediastinal masses can be challenging, and this diagnostic modality aids in distinguishing between the two. Rick et al. (52) studied 36 dogs and 24 cats using CEUS to characterize the intrathoracic masses. They performed standardized CEUS examinations on 41 pulmonary masses (68%) and 19 mediastinal masses (32%). Final diagnosis was based on cytology and/or histopathology. Absolute time to enhancement (TTE) values were recorded for the intrathoracic mass lesions and spleen, which was used as a reference parenchymal organ to calculate relative TTE (rTTE) values. The sensitivity and specificity for detecting pulmonary neoplastic masses with rTTE were 63 and 78%, respectively. The study concluded that while CEUS is a feasible method for characterizing intrathoracic mass lesions in dogs and cats, its diagnostic sensitivity for detecting neoplastic pulmonary masses was relatively low (52). Another study by Lints et al. (53) also explored the feasibility of CEUS in the diagnostic evaluation of non-cardiac thoracic disorders in small animals. They assessed 40 animals, comprising 28 dogs and 12 cats. Most neoplastic pulmonary lesions showed an inhomogeneous distribution of contrast medium, whereas inflammatory lesions displayed a homogenous distribution with typical pulmonary vessel ramification. The majority of mediastinal malignant lesions also showed an inhomogeneous distribution pattern. Lung and mediastinal abscesses had peripheral enhancement of the wall with an avascular center. The study concluded that CEUS may be a valuable tool for evaluating non-cardiac thoracic lesions. The contrast medium allows for precise delineation of lesion edges, identification of necrotic areas, and visualization of pulmonary vessel distribution. Additionally, the use of an ultrasonographic contrast medium could enhance the diagnostic usefulness of cytology and biopsy sampling (53). One needs to bear in mind that the veterinary literature is scarce regarding the use of CEUS and in the two above mentioned studies, the number of the analyzed patients are small. As much as CEUS might add some information in the staging of such patients, this diagnostic technique can be performed in the minority of the small animal practices and it also extends the time of diagnostic work up. Additionally, although it may aid in the differentiation between the inflammatory and malignant lesion, the ultimate diagnosis relies on the histological examination of the specimen.

Other imaging techniques are not routinely used in the staging of canine patients with pulmonary tumors; however, the use of positron emission tomography (PET) has been described (54). Kim et al. (55) reported the administration of 18-fluorodeoxyglucose (18FDG) as a radiotracer to evaluate a dog with pulmonary nodules. An increased 18 FDG signal was noted in all four nodules, which were confirmed as adenocarcinoma at necropsy (55). This diagnostic technique, however, is not freely available to the majority of the small animal practices therefore its used very rarely in the clinical settings.

**Figure 3:**
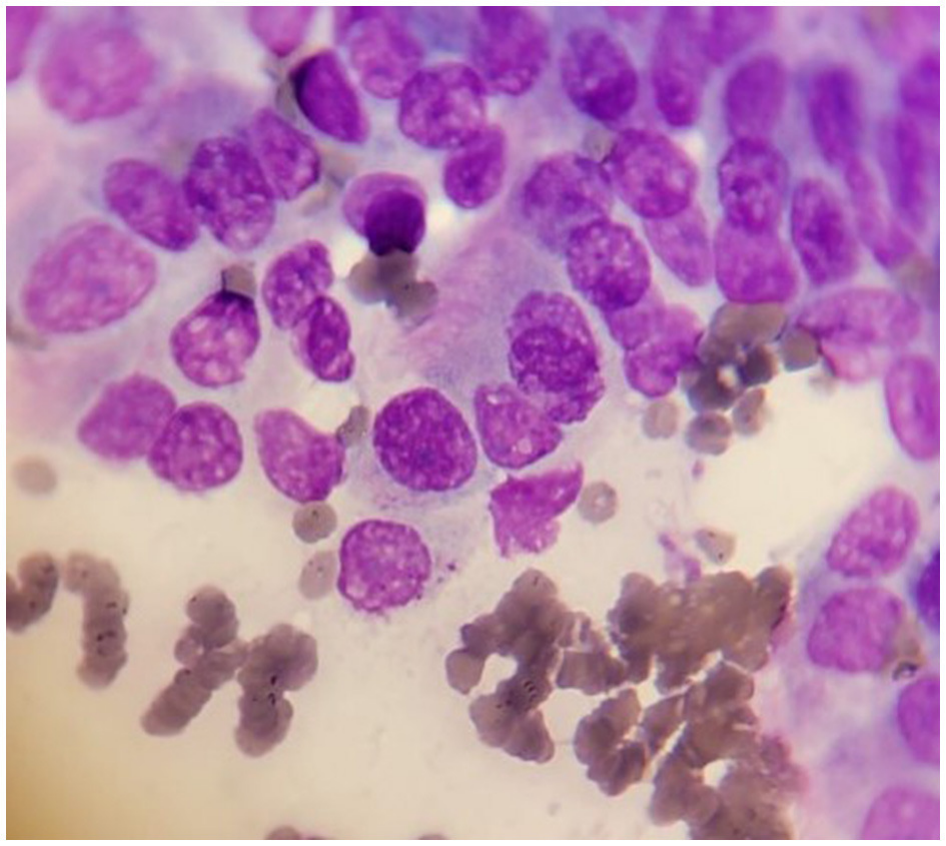
Cytology sample of well-differentiated lung carcinoma in a dog. The cells exhibit varied sizes and shapes. Some cells are larger and contain prominent nuclei. The nuclei often appear round or oval with typically stained dark purple color with 1–2 small, round nucleoli. The cytoplasm appears pale pink or light blue. In some cells is still possible to distinguish the micro-cilia.

Fine needle aspiration (FNA) biopsy is commonly used in the diagnostic workup of pulmonary masses (Figure 3). FNA is a rapid, minimally invasive, low-risk, and relatively inexpensive method for obtaining a cytologic diagnosis before planning a lung lobectomy. FNA can be performed with ultrasound, CT, or fluoroscopic guidance; however, blind aspirates have also been reported (50, 56, 57). Sedation of the patient is usually required for all these imaging modalities to prevent iatrogenic trauma while restraining the dog, especially if the dog is panting. Transthoracic ultrasound-guided FNA offers real-time visualization of the needle's path into the diseased pulmonary parenchyma, increasing the probability of obtaining a cytologic diagnosis. However, sampling is often limited to peripheral lesions adjacent to the chest wall due to potential possible interference from aerated lungs (58). As previously mentioned, CT is commonly used in veterinary medicine to evaluate pulmonary lesions, the presence of pulmonary lesions, the presence of pulmonary metastases, and regional lymphadenopathy. However, CT-guided sampling does not provide real-time visualization, is time-consuming, and can lead to increased radiation exposure for both patients and staff. Additionally, CT-guided sampling is often unsuitable for deep-seated lung lesions due to an increased risk of iatrogenic pneumothorax and/or hemorrhage.

In a study by Zekas et al. (57) evaluating 30 animals (27 dogs, two cats, and one cougar) in which CT-guided intrathoracic FNAs (12), core biopsies (10), or both (8) were performed, a diagnosis was made in 65% FNAs and 83% biopsies. Among the 18 patients with a confirmed diagnosis, the overall accuracy for diagnosis was 92% for both FNA and biopsy. The sensitivity for detecting neoplasia was 91% using FNA and 80% using biopsy. Complications were observed in 43% of the cases, including the occurrence of four instances of pneumothorax, five instances of pulmonary hemorrhage, and four cases with both. They concluded that CT-guided sampling is relatively safe and useful for diagnosing intra-thoracic lesions, especially neoplasia (57). In a separate study by Vignoli et al. (59), 52 dogs and 10 cats underwent CT-guided FNAs and tissue-core biopsies (TCB) of thoracic lesions. In this study, 95.2% of histopathological samples were diagnostic, while cytology was diagnostic in 69.4%. The general sensitivity, accuracy and PPV for FNA and TCB were 67.7, 67.7, and 100%, respectively. For TBC, these values were 96.7, 95.2, and 98.3%, respectively. Combining the two techniques, the overall mean accuracy for diagnosis was 98.4%. Complications were observed in 30.6% of the cases, with mild pneumothorax being the most common (16 cases) and mild hemorrhage occurring in only three cases. No major complications were encountered.

Very recently, transthoracic fluoroscopy-guided FNA has been described in dogs (60). Fluoroscopy offers real-time visualization of the needle trajectory through pulmonary tissue, which can improve sampling accuracy and decrease sampling time. It also allows for prompt assessment of postprocedural complications, such as pneumothorax, pulmonary hemorrhage, or hemothorax. In the study by Jacob (60), five dogs and five cats underwent fluoroscopy-guided FNAs of deep-seated pulmonary masses. Cytology results indicated carcinoma in four dogs and lymphoma in one dog. A minor postprocedural complication was noted in one dog. In cats, cytology results indicated pulmonary carcinoma in two cats, inflammation in two cats, and necrotic debris in one cat. The author concluded that this sampling technique is a safe and accurate method for obtaining cytologic samples regardless of the size and depth of the lesions. This study agrees with an earlier study by McMillan et al. (61), in which 48 FNA biopsy specimens of intrathoracic lesions were obtained. The only clinical and radiographic complication from this procedure was pneumothorax, occurring in eight dogs (20%) and resulting in one death. Definitive diagnoses were made from tissue obtained from 37 of the 48 lesions sampled, for a sensitivity rate of 77.1%. The authors concluded that FNA biopsy is a simple, safe, and accurate diagnostic technique. However, it is important to note that FNA biopsy rarely changes the treatment decision for solitary pulmonary masses based on CT findings, as surgical resection is the treatment of choice for most solitary solid tumors.

Endoscopic bronchoalveolar lavage is not recommended for diagnosing lung tumors in dogs and is rarely performed as part of the investigation procedures in these patients (61).

Pretreatment lung biopsy is not commonly performed, and its clinical relevance is questionable (62). It may be considered in cases where multiple attempts in diagnosis by FNA have failed, or in rare situations where histologic grade and/or degree of differentiation is required before surgery, such as when the owner is deciding about lung lobectomy or when there is uncertainty about whether the pulmonary mass is primary or part of systemic/metastatic neoplasia. However, clinicians must be aware that in a large proportion of dogs undergoing tru-cut biopsies of lung masses, the grade cannot be assessed due to the small and often unrepresentative sample. A lung tissue biopsy can be obtained by needle biopsy, bronchoscopy, keyhole incision with staple application, and thoracoscopy (7, 8, 57, 62, 63). In a study of dogs and cats undergoing CT-guided tissue core biopsies of intrathoracic lesions, the diagnostic accuracy was 92%, and the sensitivity for diagnosing neoplasia using tissue core biopsies was 80%. The complication rate was fairly high (43%), with the most common being pulmonary hemorrhage (30%) and pneumothorax (27%) (57). Bronchoscopic lung biopsy in dogs with lung neoplasia has also been described in the literature, albeit with rare success. Bronchoscopic findings in canine patients with pulmonary neoplasia include narrowing of bronchi, mucosal erosions, and mucosal swelling and hyperemia (64). The keyhole lung biopsy technique has also been reported but primarily in non-neoplastic lung lesions in dogs. In this technique, a small thoracotomy window is made and the lung vessels and small airways are sealed off using a surgical stapler (63).

In a study by Wormser et al., 11 dogs and cats underwent thoracoscopic-assisted pulmonary surgery for partial and complete lung lobectomy as means of diagnostic technique. No intraoperative complications were encountered and all patients were discharged home, with nine out of 11 alive 6 months after surgery. This study confirms that this surgical technique can be safely performed as part of the diagnostic plan in animals (65). In another study, thoracoscopy was used to determine the cause of pleural effusion in 18 cats and dogs. Biopsies of several intrathoracic lesions were performed and neoplasia was diagnosed as a cause of the pleural effusion in eight cases (66). For all of the aforementioned techniques for collecting pretreatment lung biopsy samples, the animal needs to be sedated or anesthetized.

Differentiating poorly differentiated primary lung tumors from metastatic lesions can occasionally be difficult but is critical due to differences in prognosis and treatment. Differentiating multiple primary lung carcinomas from metastatic carcinoma of a distant-located primary or metastases of unknown origin can be challenging. In humans, a combination of immunohistochemical (IHC) markers, including thyroid transcription factor-1 (TTF-1), Napsin A, surfactant protein-A (SP-A), is used to diagnose carcinomas of pulmonary origin (67–72). TTF-1 is a nuclear thyroid transcription factor expressed in normal lung and thyroid and is detected in 70% of human lung tumors (69–72). Napsin A is detected in 70–90% of human lung tumors, with higher expression associated with longer survival (68–70, 73, 74). Immunoreactivity of SP-A is observed in surfactants, type II pneumocytes, and in 45–60% of human pulmonary carcinomas (74, 75).

Of the antibodies used in humans, TTF-1 is the best characterized and studied in dogs to differentiate lung carcinoma from another type of metastatic carcinomas (76). However, up to 36% of canine pulmonary carcinomas lack TTF-1 immunoreactivity, and in addition, thyroid carcinoma, which frequently metastasizes to the lungs, can be immunohistochemically positive in up to 80% of cases (76, 77). Napsin A immunoreactivity was detected in 5/5 of canine pulmonary carcinomas, normal type II pneumocytes, and some bronchial epithelial cells (78). In humans, this marker stains over 90% primary pulmonary carcinomas, with 100% reactivity in adenocarcinomas and no reactivity in squamous cell carcinomas (79). In dogs, Napsin A is expressed in 62% of thyroid carcinomas and in around 60% of renal cell carcinomas (78, 80).

In a study assessing the immunohistochemical expression of TTF-1 in 34 primary and 27 non-primary canine lung tumors, TTF-1 was 100% specific and 85% sensitive for primary lung carcinoma. Metastatic tumors were always negative. The tumors scoring negative were squamous cell carcinoma and papillary carcinoma. There was no significant relationship betweenv the percentage of labeled tumor cells and clinicopathologic parameters, such as age, gender, histological type, tumor grade, TNM stage, nodal status, and Mindbomb Homolog-1 (MIB-1) index (81). Beck et al. (82) compared the immunohistochemical expression of SP-A and Napsin A with TTF-1. They examined 67 formalin-fixed, paraffin-embedded canine pulmonary tumors. Only 3% of lung tumors were negative for SP-A, compared with 7 and 9% for Napsin A and TTF-1, respectively. Each antigen was detected in a greater percentage of cells in tumors with acinar or papillary patterns compared to those with squamous differentiation. They concluded that SP-A and Napsin A are useful markers for canine lung epithelial neoplasia and that SP-A is the most sensitive and specific. SP-A can be used in combination with TTF-1 or Napsin A to improve the detection and differentiation of pulmonary carcinomas from metastatic tumors in the lungs (82).

Canine lung cancer resembles human lung cancer in never-smokers. Human never-smoker lung cancer is primarily non-small cell lung cancer (NSCLC), which accounts for 10–25% of cases and is characterized by a high incidence of gene mutations, such as those affecting EGFR. NSCLC arises from the lung tissue, unlike small cell lung cancer (SCLC), which originates from the bronchi and is more common in smokers. The two main histological types of NSCLC are adenocarcinoma (AC) and squamous cell carcinoma (SCC). The etiology of never-smoker lung cancer in humans is distinct from that of smokers and is associated with factors such as environmental exposure (secondhand smoke, radon, asbestos, arsenic, silica, and pollution), as well as age, sex, family history and genetics (83). Unique genomic characteristics of human never-smoker lung cancer include somatic activating point mutations or fusions affecting EGFR (45%), anaplastic lymphoma kinase (ALK; 5–11%), ROS (1.5–6%), human epidermal growth factor receptor 2 (HER2; 3–5%) and rearranged during transfection (RET; 2%) (84). Survival and outcomes in humans with lung cancer depend on the molecular subtype and treatment. EGFR inhibitors improve outcomes in EGFR-mutant lung cancers; however, 85% of never-smoker-lung AC and SCC in the USA are EGFR wild-type.

In contrast to humans, Mariotti et al. (85) found that EGFR mutations, overexpression, or phosphorylation were rare in canine pulmonary adenocarcinoma compared with matched non-affected chemotherapy-naive lung tissue. They also documented significant overexpression and/or phosphorylation of the platelet derived growth factor receptor alfa (PDGFRα), ALK, and HER2 (85). However, another study by Sabatini et al., reported that 73% of analyzed tumors were EGFR-positive, suggesting a possible correlation with a negative prognosis (86). Additionally, higher EGFR expression was observed in lungs presenting anthracosis, indicating a potential link between EGFR overexpression and air pollution-related carcinogenesis. Lorch et al. (87) discovered somatic, coding HER2 point mutations in 38% of canine pulmonary adenocarcinomas but none in adenosquamous or SCC. The majority (93%) of HER2 mutations were hotspot V659E transmembrane domain (TMD) mutations, comparable to activating mutations at this same site in human cancer. HER2V659E mutations were detected in the plasma of 33% of dogs with localized HER2V659E tumors. Canine pulmonary adenocarcinoma cell lines exhibited constitutive phosphorylation of AKT and significantly higher sensitivity to HER2 inhibitors, such as lapatinib and neratinib, compared to HER2-wild-type cell lines. This study represents a significant step toward a molecular understanding of and drug development for canine lung carcinoma (87). Another small study using IHC also found almost 70% expression of HER-2 in canine lung carcinoma (88).

### Treatment and prognosis

**Figure 4:**
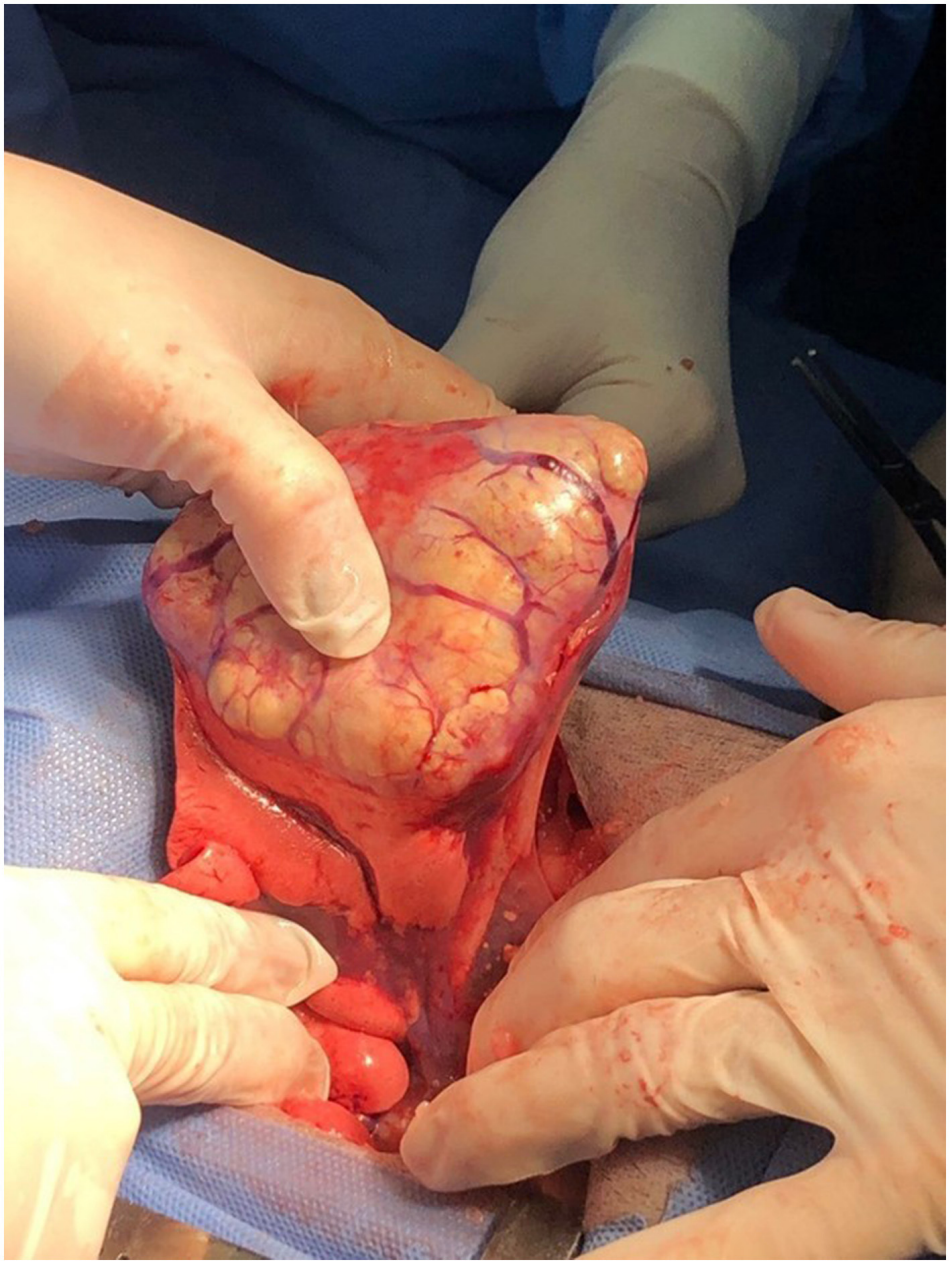
Surgical resection of a lung carcinoma. The intercostal approach for a lung lobectomy in a dog with a well-differentiated lung carcinoma.

**Figure 5:**
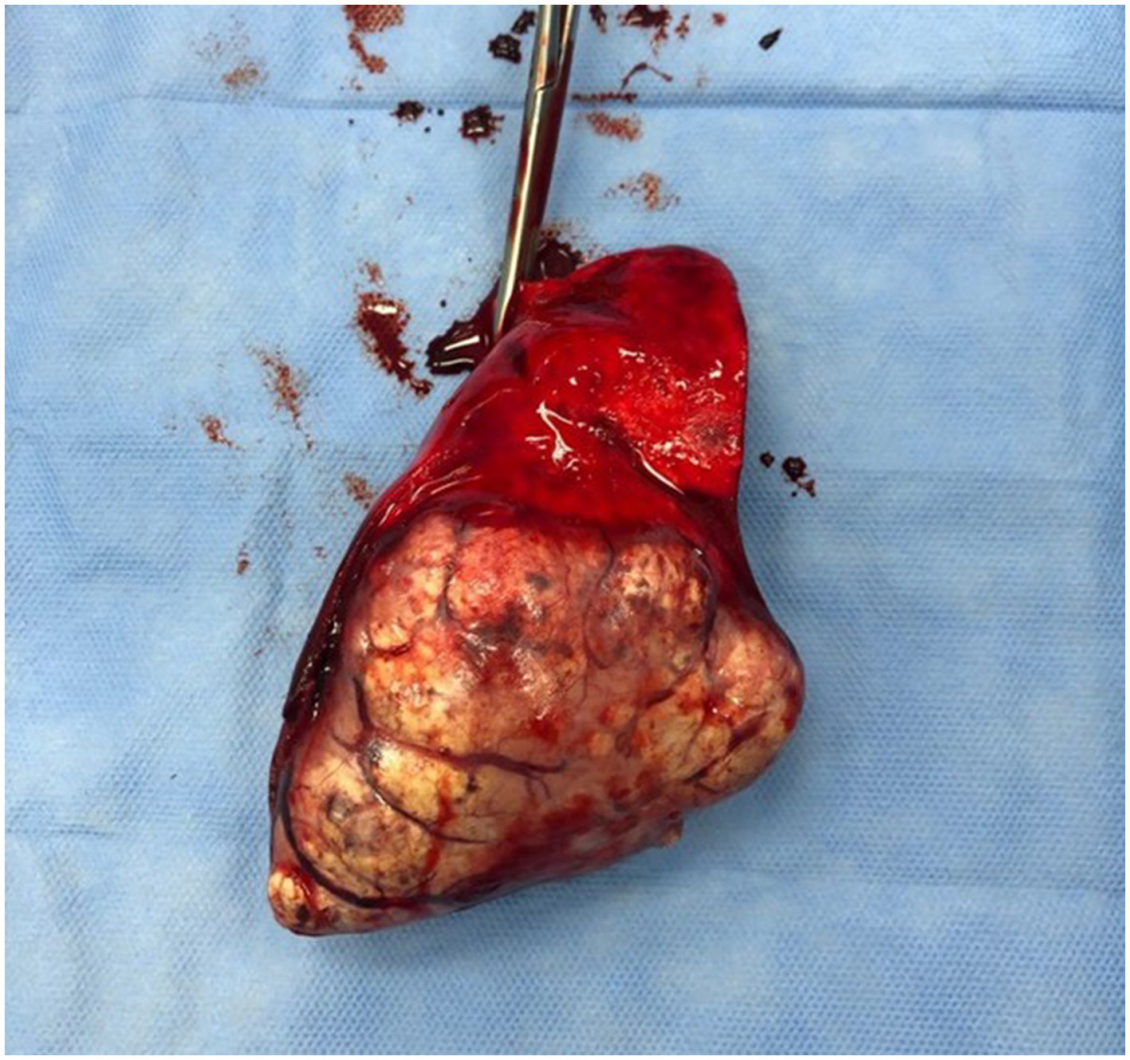
The gross appearance of a well-differentiated lung carcinoma post-surgical resection as in the previous picture.

Surgery is the preferred treatment for dogs with primary pulmonary tumors (89–92). The surgical approach is determined by the clinician and the tumor location within the lung. For unilateral tumors, a lateral thoracotomy is preferred, although a median sternotomy can also be used (Figures 4, 5). Thoracoscopic lung lobectomy is indicated for peripherally located tumors that are in a suitable location for this minimally invasive technique. However, this approach requires a surgeon with advanced training in minimally invasive surgery. If the lung tumor spreads across multiple lobes, a sternal thoracotomy is advisable. The procedures typically involve removing all or part of a lung lobe as well as performing lymphadenectomy. In most cases, a complete lung lobectomy is indicated and performed. However, a partial lobectomy can be performed for small, peripherally located tumors or for pulmonary meta-stasectomy. The patient's position during surgery is adjusted based on the tumor's location to provide optimal access to the mass. The surgical procedure is commonly performed through the fourth or fifth intercostal space.

Complete or partial lung lobectomy can be performed using either suturing methods or surgical staplers (64). Surgical staples are considered the technique of choice for partial and complete lung lobectomy due to their safety, speed, and efficiency, with minimal side effects for the patients (21, 89, 90). However, a recent study on canine cadavers investigating different sealing methods after partial pulmonary lobectomy found that surgical glue was superior to interlocking sutures and stapling devices at supraphysiological levels of ventilatory pressure. Therefore, in some of these patients, surgical glue could possibly be a safer and more cost-effective sealing method compared to surgical staplers (91). However, its use in such patients requires confirmation on the live patients.

In a study by Mayhew et al., 22 medium to large breed dogs underwent video-assisted thoracoscopic surgery (VATS) for lobectomy of lung tumors and were compared to a group of 24 dogs who underwent lung lobectomy via an open thoracotomy (OT) (92). There were no significant differences between the VATS and OT regarding most variables, although surgery time was significantly longer for VATS than for OT. In this study, an endoscopic stapler was used for lung lobectomy via either 3-or 4-port technique.

In a study by Bleakley et al. (93), 13 dogs underwent thoracoscopic lung lobectomy. The authors compared the long-term outcomes between dogs with primary lung tumors treated with lobectomy via thoracoscopy or thoracotomy. Three thoracotomies were converted to OT due to poor visualization. There were no differences in short-term outcomes between dogs that had thoracoscopy and those that were converted to OT, concluding that selected primary lung tumors in dogs can be safely resected with thoracoscopy without affecting long-term survival (93). Another study examined nine dogs with either primary or metastatic pulmonary tumors, where thoracoscopic lung lobectomy was successfully performed in five dogs, while four dogs required conversion to OT due to poor visibility. The authors suggested that cases suitable for thoracoscopic lung lobectomy were those with masses located distant from the hilus. Cranial lung lobectomy was easier than caudal lung lobectomy but both could be performed successfully (94). In an earlier study by Walsh et al. comparing both techniques, dogs that had a pericardiectomy performed via VATS showed less evidence of postoperative pain and lower analgesic requirements compared with dogs that had a pericardiectomy performed via OT (95). A more recent study assessed complications and outcomes of thoracoscopic-assisted lung lobectomy in dogs (96). The study involved 30 dogs, with 12 intraoperative complications recorded in 11 dogs, and six dogs required conversion to OT. Reasons for conversion included adhesions, difficulty manipulating the lesion through mini-thoracotomy, and acute oxygen desaturation. Complications were documented in eight dogs before discharge, with five of these being classified as mild. Overall the procedure resulted in few major complications (96). From the available veterinary literature, conversion rates for thoracoscopic lung lobectomy range between 9 and 23%, with the most common reason for conversion being poor visualization (94, 97–99). To improve visualization in these patients treated by thoracoscopy, one lung ventilation should be considered (94, 97–99). It is important to biopsy or remove the tracheobronchial lymph nodes during the surgery, as metastatic disease to the lymph nodes significantly affects prognosis (45).

In humans, chemotherapy is considered a standard of care in the treatment of lung cancer and is administered either in an adjuvant or palliative setting (100). Chemotherapy is similarly used in dogs with pulmonary carcinoma in both settings; however, unlike in humans, little is known about its efficacy. Chemotherapy in dogs with macroscopic disease has been largely unrewarding. Several earlier studies have assessed the efficacy and safety of various chemotherapeutic agents such as doxorubicin, mitoxantrone, vindesine, and cisplatin (21, 101, 102) (Table 3). In the doxorubicin study, only one dog with papillary pulmonary adenocarcinoma was included, and this dog experienced progressive disease despite the treatment with doxorubicin (101). In another study, three dogs diagnosed with lung adenocarcinoma showed no response to mitoxantrone treatment (102). Separately, two dogs treated with vindesine had minimal response, while two dogs treated with a combination of vindesine and cisplatin showed over a 50% reduction in measurable disease (21). The response and results of the three above studies are not encouraging, however the number of dogs examined are very small making it difficult to base the clinical decision and judgment.

**Table 3:** Table summarizing the chemotherapy agents described in the veterinary literature and used in the treatment of the lung carcinoma in dogs.

**Chemotherapy**	**Disease**	**Response**	**References**
Doxorubicin (30 mg/m^2^ IV)–2 doses every 21 days	Macroscopic disease, various neoplasms (one dog with pulmonary adenocarcinoma available for evaluation)	Progressive disease (PD) (1/1)	(101)
Mitoxantrone (2.5–5 mg/m^2^ IV)–1–10 doses every 21 days	Macroscopic disease, various neoplasms (three dogs with pulmonary adenocarcinoma)	No measurable response (3/3)	(102)
Vindesin	Macroscopic disease (*n* = 2)	Minimal response (2/2)	(21)
Vindesin + Cisplatin	Macroscopic disease (*n* = 2)	Partial response (PR) > 50% (2/2)	
Vinorelbine (10–20 mg/m^2^ IV)–1–11 doses every 7 days	Macroscopic disease (7 dogs with bronchoalveolar carcinoma)	PR(2/7)	(103)
	Adjuvant (three cases of bronchoalveolar carcinoma)	Individual survival times of 113, 169, and >730 days	
Vinorelbine (15 mg/m^2^ IV)–4 doses 7 days apart and then every 14 days until disease progression	Macroscopic disease (10 dogs with stage IV pulmonary carcinoma)	PR (8/10 with a median time to progression of 88 days) MST of 100 days.	(104)
Carboplatin (200–250 mg/m IV) + Bleomycin (15–20 UI/m2 IV)–1–6 cycles every 21 days	Macroscopic disease (three dogs with advanced lung carcinoma)	Stable disease (SD) (2/3 with survival time of 180 and 227 days); PD (1/3 with survival time of 114 days)	(105)
Metronomic chemotherapy with cyclophosphamide (10 mg/m^2^ every 24–48 h) + piroxicam (0.3 mg/kg every 24 h) + Thalidomide (2 mg/kg every 24 h)	Macroscopic disease (25 dogs with advanced primary lung cancer)	PR (4/25); SD (19/25); PD (2/25). Median time to progression was 172 days and median survival time 139 days	(23)^*^
Maximum tolerated dose chemotherapy with vinorelbine (15–18 mg/m^2^ IV every 7 days), carboplatin (300 mg/m^2^ IV every 21 days), or gemcitabine (800 mg/m^2^ IV every 7 days)	Macroscopic disease (11 dogs with advanced primary lung cancer)	PR (1/11—dog treated with vinorelbine); SD (3/11); PD (7/11)	
Surgery alone	No systemic treatment (36 dogs with advanced primary lung cancer, 28 survived immediate post-operative period)	Median time to progression of 110 days and survival time of 111 days	
Palliative care with Prednisone or NSAIDs (meloxicam or piroxicam)	Macroscopic disease (19 dogs with advanced primary lung cancer)	Median time to progression of 20 days and survival time of 60 days	

^*^Statistical significant differences found on the median time to progression and survival time between the metronomic group and the other three groups separately (p < 0.001) (23).

Further studies explored the use of vinorelbine. In one study, seven dogs with pulmonary cancer were assessed. Two dogs with measurable bronchoalveolar carcinoma achieved a partial response following treatment with vinorelbine. Three additional dogs with microscopic disease received adjuvant treatment with vinorelbine, resulting in individual survival times of 113, 169, and >730 days (103). A more recent study treated 10 dogs with stage IV pulmonary carcinoma using vinorelbine, resulting in eight dogs achieving a partial response. The median time to progression was 88 days (range 7–112 days), and the median survival time (MST) for all dogs was 100 days (range 7–635). The most common side effect reported was neutropenia, but overall vinorelbine treatment was well-tolerated. Despite the small number of published studies and the small number of included patients, partial responses in both the macroscopic and microscopic setting are promising and bleomycin may be considered a first-line treatment for advanced lung tumors (104).

A combination of carboplatin and bleomycin has also been used in the treatment of various canine carcinomas (105). Carboplatin is generally well-tolerated in dogs; however, mild myelotoxicity and gastrointestinal side effects are fairly common. Bleomycin, an antibiotic used in human medicine, is mainly used as an adjuvant therapy for various cancers due to its lower myelotoxicity compared with other chemotherapy agents. As a single agent, bleomycin has modest activity due to its low cellular membrane permeability and low intracellular concentration (106). The main side effects of bleomycin in human and animal models are pneumonitis and pulmonary fibrosis, typically seen at cumulative doses higher than 400.000 IU/m^2^. The addition of bleomycin to carboplatin leads to DNA breaks, leading to cancer cell apoptosis (107, 108). The combination of these two medications, due to their potential synergistic effects, could be beneficial with a lower rate of adverse events. In the study by Giuliano et al. (105), 30 canine patients were assessed. Among them, three dogs had pulmonary adenocarcinoma. Two of these three patients achieved stable disease, while one experienced progressive disease (105). The reader must bear in mind this is one study with a very small number of patients, however these results are encouraging and this drug combination could be used in the management of canine pulmonary carcinoma.

Metronomic chemotherapy is a treatment involving frequent, low-dose oral administration of a chemotherapy drug, delivered continuously without prolonged drug-free intervals (109, 110). Metronomic chemotherapy is a potential tool for treating various cancers in both people and animals (111, 112). The primary target of this therapy is endothelial cells involved in angiogenesis. Additionally, it also efficiently targets the heterogenous population of tumor cells, elicits innate and regulatory T cells, and exerts immunomodulatory effects in tumor microenvironment (113). The therapeutic benefits of metronomic chemotherapy have been observed in human cancer patients where conventional chemotherapy was no longer effective. Notably, positive results have been seen in individuals with metastatic breast cancer, NSCLC, and colorectal carcinoma (114).

The management of advanced primary lung carcinoma with metronomic chemotherapy has also been described in dogs. In a study by Polton et al. (23), 91 dogs were included: 25 received metronomic chemotherapy (cyclophosphamide, piroxicam, and thalidomide), 36 underwent surgical treatment, 11 were treated with maximum-tolerated dose (MTD) chemotherapy, and 19 received no treatment. Quality of life improved in dogs receiving metronomic chemotherapy. The median time to progression was significantly longer for patients receiving metronomic chemotherapy (172 days) compared to those undergoing surgery (87 days), receiving MTD (22 days), or receiving no oncological treatment (20 days). Median survival time was also longer in patients receiving metronomic chemotherapy (139 days) than in those undergoing surgical treatment (92 days), receiving MTD (61 days), or receiving no treatment at all (60 days). Dogs not receiving metronomic chemotherapy had a 2.6-fold increased risk of tumor progression and a 2.7-fold increased risk of death. The authors concluded that in advanced canine primary pulmonary carcinoma, metronomic chemotherapy can achieve a measurable clinical benefit without significant risk or toxicity, making this treatment a potential alternative to other therapeutic approaches (23). Adjuvant maximum tolerated doses of lomustine provided increased survival times (374–568 days) for dogs with solitary lung histiocytic sarcoma (115). This is the biggest study evaluating the highest number of patients with pulmonary carcinoma, including 25 dogs with macroscopic disease. Partial remission and stable disease were achieved in 4 and 19 cases, respectively. The results, the ease of administration of this therapy as well as the low toxicity profile, make this treatment very attractive.

There are reports in veterinary medicine about the delivery of aerosolized chemotherapy or cytokines in dogs with either primary or metastatic pulmonary neoplasia. This treatment appears to be well-tolerated in dogs, achieving complete or partial responses in metastatic tumors and stable or progressive disease in primary lung tumors (116, 117). In a palliative setting, intrapleural chemotherapy has been used to reduce the volume of pleural effusion in dogs with carcinomatosis, sarcomatosis, or mesothelioma. Carboplatin or mitoxantrone can also be administered, using doses similar to those employed in the intravenous maximum tolerated chemotherapy, but with further dilution. This chemotherapy delivery is rarely used in the management of dogs with pulmonary carcinoma. Studies on these are scarce therefore meaningful conclusions and therapeutic effects are difficult to make (118).

Targeted therapy and immunotherapy are commonly used in people with lung cancer. EGFR mutations are common in NSCLC in humans, and targeted treatments with erlotinib and gefitinib have been used for decades (119). In contrast, HER2 mutations are uncommon in people with lung cancer, found only about 3% of human patients (120). HER2 amplification and protein overexpression have been reported in various human neoplasms, particularly in breast, and gastric cancers, but also in lung cancer (88, 121–123). HER2 serves as both a diagnostic marker and a therapeutic target in these tumors in people. HER2-targeted therapies, such as lapatinib and trastuzumab, show clinical responses in humans with HER2-positive cancers. HER2 is overexpressed in a proportion of human patients with NSCLS and is related to its tumorigenesis (124). HER2 protein gene amplification and overexpression are observed in up to 35% of human patients with lung NSCLC, while HER2 mutations are seen in only 3% of patients (124). The role of HER2-targeted treatment remains controversial in humans; however, trastuzumab and other HER2-targeted therapies have shown some results in NSCLC (125–127). Yoshimoto et al. (128), evaluated HER2 expression in six canine pulmonary lung tumors (carcinomas) using immunohistochemistry and compared it to healthy lung tissue. Immunohistochemical analysis revealed that 19% of samples scored 3+, 50% scored 2+, 31% 1+, and 0% scored 0. Overall, 69% of the primary lung tumor tissues were HER2 positive, scoring ≥2. This study suggests that HER-2-targeted therapy and HER-2-targeting recombinant Listeria vaccines could have some therapeutic anti-tumor effects in the case of canine primary lung carcinoma. This finding prompts further evaluation of the role of HER2 in canine pulmonary tumors as a mechanism of malignancy and as a therapeutic target (129). It also opens up the possibility of using targeted therapy in the management of canine pulmonary cancer.

Lapatinib, a dual inhibitor of EGFR and HER2, has not yet been used in the treatment of lung carcinoma in dogs. However, its use in canine patients with muscle-invasive urothelial carcinoma has shown positive results, particularly in dogs with HER2 overexpression, and safe profile at a dose of 20–30 mg/kg, along with piroxicam at 0.3 mg/kg, both administered orally once a day (130). On the other hand, toceranib phosphate, a tyrosine-kinase inhibitor, licensed for use in dogs, is primarily recommended for the treatment of canine mast cell tumors due to its inhibition of the receptor KIT. Toceranib phosphate is also capable of blocking PDGFR, VEGFR, Flt-3, CSF1R, RET, ALK, and AXL, which may provide potential benefits for dogs with primary lung cancer. In a phase I study, monotherapy with toceranib resulted in stable disease for more than 10 weeks in a dog with primary lung carcinoma (131).

Locally advanced human NSCLS is routinely treated with concurrent chemo-radiotherapy. However, despite this intensive treatment strategy and its risk of severe side effects, 2-year progression-free survival remains low at 20–30%, due to a lack of tumor control (132, 133). Radiotherapy has been suggested to prolong the survival of medically inoperable patients or those refusing surgery, including those with stage I/II disease (133). The role of radiotherapy in dogs with solitary pulmonary carcinoma is still controversial. In a retrospective study, Kawabe et al. (134) described the effects of hypofractionated radiotherapy in nine dogs with solitary lung adenocarcinoma that were later considered for surgical resection. Acute and late radiation-induced toxicity to the skin and/or lungs developed in all nine dogs, but these effects were self-limiting or improved with short-term anti-inflammatory treatment. The median interval between the last radiotherapy session and maximum tumor size reduction was 56 days. Tumor progression after initial size reduction was documented in three dogs. Seven of the nine dogs underwent lobectomy, and five received systemic chemotherapy concurrent with or after radiotherapy. This study suggested that hypofractionated radiotherapy is useful in treating large primary lung tumors (134).

Stereotactic body radiation therapy (SBRT) utilizes high, precisely delivered doses of radiation administered over a short period to tumors as an alternative to surgery. With SBRT, a more conformal radiation approach and less toxicity are anticipated. In humans, this type of radiotherapy is usually used in the treatment of small lesions (e.g., liver); however, it has also proven to be a safe, effective, and efficient treatment for NSCLC and is considered the standard of care for medically or functionally inoperable patients (135).

In a study by Wormhoudt Martin et al. (136), 21 primary pulmonary carcinomas in 19 dogs were treated with SBRT for local tumor control. The overall MST was 343 days, with 38% of patients alive at 1 year. Twenty-eight percent of dogs experienced acute lung VRTOG effects, and 17% experienced late lung VRTOG effects. The best response included a complete response in 17% of cases, a partial response in 42%, and stable disease in 42%. Progressive disease was noted in seven dogs 229 days after SBRT. Overall MST was 343 days (range 46–905) with 38% alive at 1 year. The median progressive-free survival (PFS) was 238 days (range 72–751 days), and the disease-specific survival was 343 days (range 46–751 days). This study documents that SBRT is a safe and effective alternative to surgery and could be considered an option for dogs with stage 3 and 4 lung tumors (136).

Immunotherapy, especially targeting checkpoint inhibitors, such as programmed cell death protein-1/programmed cell death protein-ligand 1 (PD1/PD-L1) and cytotoxic T-lymphocyte associated protein 4 (CTLA4), is often used in people with NSCLC. Nivolumab, an anti-PD1 monoclonal antibody has been approved for PD-L1 IHC-positive metastatic NSCLC in humans (137). Immune checkpoint inhibitor (ICI) treatment in dogs is still in its infancy, but various studies have been published suggesting potential applications of ICI in dogs, and various canine ICIs are under investigation (138).

Several prognostic factors influence the survival of dogs with primary pulmonary neoplasia. These include lymph node status, tumor stage, histologic type, grade, presence of clinical signs, and tumor volume/size. A diagnosis of lymph node-positive primary canine lung cancer carries a grave prognosis, with a median survival time of 26–167 days, compared to 285–452 days for lymph node-negative cases (4, 8, 9, 45, 139). Dogs with solitary lung tumors (T1 clinical stage) have an MST of 790 days, significantly longer than dogs with multiple lung tumors (T2 clinical stage) at 196 days, and dogs with lung tumors invading adjacent structures (T3 clinical stage) at 81 days (8). In a study by Polton et al. (140), dogs with papillary adenocarcinoma and a clinical stage T1N0M0 had a MST of 555 days, significantly longer than dogs with any other tumor type, which had an MST of 72 days (140). Dogs presenting with clinical signs had an MST of 240 days, compared to 545 days for asymptomatic dogs (8). A very recent study by Ichimata et al. (141) reported that age was associated with overall survival, with a 1.28-fold increased risk of death for each additional year. In that study the PFI and OST were 754 and 716 days, respectively (141).

## Conclusions and future perspectives: a 5-year horizon

Over the next 5 years, significant advancements in the diagnosis and treatment of canine pulmonary carcinoma are anticipated. These developments will likely improve the outcomes and quality of life for affected dogs. The use of advanced diagnostic imaging techniques, such as thoracic computed tomography and positron emission tomography, is expected to become more widespread. These technologies provide precise tumor localization and staging, enhancing treatment planning and prognosis accuracy (41, 45, 55). Moreover, molecular profiling will play a crucial role in identifying genetic mutations and molecular targets within canine tumors. This approach will enable the development of personalized treatment plans, similar to human oncology practices (67–72).

Minimally invasive surgical techniques, including thoracoscopic lung lobectomy, are likely to gain broader acceptance due to their reduced morbidity and quicker recovery times (92–94). Improvements in surgical tools and techniques will increase the success rates of these procedures, making them viable options even for more complex cases. Thoracoscopic-assisted pulmonary surgery has already shown promise in terms of safety and efficacy, and its use is expected to expand (96).

Metronomic chemotherapy, which involves the frequent, low-dose administration of chemotherapeutic agents, has shown promise in improving outcomes with fewer side effects (109). Ongoing research will refine these protocols and identify optimal drug combinations, potentially establishing this as a standard approach for treating various stages of canine pulmonary carcinoma (23, 111, 112). Studies have indicated that metronomic chemotherapy can significantly prolong survival times and improve the quality of life in dogs with advanced primary lung carcinoma (23).

Targeted therapies, such as those inhibiting EGFR and human epidermal growth factor receptor 2 (HER2), and immunotherapies like checkpoint inhibitors (PD-1/PD-L1, CTLA-4), are on the horizon for canine patients (88, 119–124). Clinical trials and ongoing research are expected to provide new, effective treatment options for dogs with primary lung tumors. The expression of HER2 in canine pulmonary tumors suggests that HER2-targeted therapies could have therapeutic potential (128). Similarly, toceranib phosphate, a tyrosine kinase inhibitor, has shown promise in managing primary lung cancer in dogs (131).

The future of veterinary oncology is moving toward personalized medicine, where treatments are tailored to the individual genetic and molecular profile of each tumor. This approach, already gaining traction in human medicine, will enable more precise and effective therapies, potentially improving survival rates and quality of life for canine patients (87). Research into molecular characteristics of canine lung cancer, such as the identification of HER2 mutations, will drive the development of targeted therapies (87, 88). Advancements in supportive care will be crucial in managing side effects and complications from cancer treatments. Improved pain management, nutritional support, and holistic care approaches will focus on the overall wellbeing of the patient. This comprehensive approach will ensure that dogs undergoing cancer treatment maintain a better quality of life (21, 89, 90).

Continued collaboration between veterinary oncologists, researchers, and pharmaceutical companies will drive the development of new therapies and improve existing ones. Enhanced funding and support for veterinary oncology research will be essential in bringing these innovations to clinical practice. Research into the efficacy of immunotherapies and targeted treatments, such as nivolumab and canine-specific immune checkpoint inhibitors, will be particularly important (137, 138). These advancements collectively hold the promise of significantly improving the prognosis and quality of life for dogs diagnosed with primary pulmonary neoplasia.

## Conclusion

Dogs with suspected primary pulmonary neoplasia must be approached systematically to ensure the best of care. This includes regular health checks and screening thoracic radiographies for middle-aged to older dogs presenting with cough or respiratory signs to improve early detection of lung cancer. Thoracic CT remains the diagnostic imaging modality of choice for staging and surgical planning. Surgery with lung lobectomy is the most effective treatment, resulting in the longest survival times.

Adjuvant treatment with conventional or metronomic chemotherapy remains controversial but can offer some other benefits to some patients. Emerging targeted therapies and immunotherapies are likely to provide new effective treatment options in the near future.
